# Carbon dioxide valorization into resveratrol via lithoautotrophic fermentation using engineered *Cupriavidus necator* H16

**DOI:** 10.1186/s12934-024-02398-x

**Published:** 2024-04-27

**Authors:** Yongjae Jang, Yeon Ji Lee, Gyeongtaek Gong, Sun-Mi Lee, Youngsoon Um, Kyoung Heon Kim, Ja Kyong Ko

**Affiliations:** 1 Clean Energy Research Center, Korea Institute of Science and Technology (KIST), Seoul, 02792 Republic of Korea; 2 Division of Energy and Environment Technology, KIST School, University of Science and Technology, Seoul, 02792 Republic of Korea; 3 Department of Biotechnology, Korea University, Seoul, 02841 Republic of Korea

**Keywords:** CO_2_ valorization, *Cupriviadus necator* H16, Lithoautotrophic production, Resveratrol

## Abstract

**Background:**

Industrial biomanufacturing of value-added products using CO_2_ as a carbon source is considered more sustainable, cost-effective and resource-efficient than using common carbohydrate feedstocks. *Cupriavidus necator* H16 is a representative H_2_-oxidizing lithoautotrophic bacterium that can be utilized to valorize CO_2_ into valuable chemicals and has recently gained much attention as a promising platform host for versatile C1-based biomanufacturing. Since this microbial platform is genetically tractable and has a high-flux carbon storage pathway, it has been engineered to produce a variety of valuable compounds from renewable carbon sources. In this study, the bacterium was engineered to produce resveratrol autotrophically using an artificial phenylpropanoid pathway.

**Results:**

The heterologous genes involved in the resveratrol biosynthetic pathway—tyrosine ammonia lyase (*TAL*), 4-coumaroyl CoA ligase (*4CL*), and stilbene synthase (*STS*) —were implemented in *C. necator* H16. The overexpression of acetyl-CoA carboxylase (*ACC*), disruption of the PHB synthetic pathway, and an increase in the copy number of *STS* genes enhanced resveratrol production. In particular, the increased copies of _*Vv*_*STS* derived from *Vitis vinifera* resulted a 2-fold improvement in resveratrol synthesis from fructose. The final engineered CR-5 strain produced 1.9 mg/L of resveratrol from CO_2_ and tyrosine via lithoautotrophic fermentation.

**Conclusions:**

To the best of our knowledge, this study is the first to describe the valorization of CO_2_ into polyphenolic compounds by engineering a phenylpropanoid pathway using the lithoautotrophic bacterium *C. necator* H16, demonstrating the potential of this strain a platform for sustainable chemical production.

**Supplementary Information:**

The online version contains supplementary material available at 10.1186/s12934-024-02398-x.

## Background

The increasing concerns regarding global warming and the depletion of fossil fuel resources have prompted researchers to explore sustainable methods for chemical production from renewable carbon resources. Microbial fermentation has emerged as a promising approach, aided by the recent advancements in synthetic biology-guided metabolic engineering tools [1]. Polyphenolic compounds, including resveratrol, bisdemethoxycurcumin, and naringenin, have been extensively studied due to their diverse biological activities [2]. Resveratrol (*trans*-3,5,4′-transhydroxy-stilbene), a naturally occurring stilbene, has been widely used in various applications, including as a flavoring, fragrance, medicinal, and nutritional supplement [3, 4]. Commercially, these bioactive compounds are either extracted from plants such as grapes, berries, peanuts, and other vine plants in trace amounts or synthesized chemically [3, 5]. Owing to their health benefits and the increasing demand, the microbial production of plant-derived natural products has mainly been achieved by microbial metabolic engineering. Considering the number of benefits such as low production cost, high product purity, and sustainability, microbial fermentation process of resveratrol production provides a promising alternative to plant extraction or chemical synthesis [3, 6].

Resveratrol is synthesized in plants via the phenylpropanoid pathway, beginning with the synthesis of phenylpropanoids (such as coumaric and ferulic acids) from the aromatic amino acids phenylalanine and tyrosine [2, 7]. Phenylpropanoids are converted into various polyphenolic compounds via type III polyketide synthases (PKS). PKS include stilbene synthase for the synthesis of stilbenes such as resveratrol and chalcone synthase for the synthesis of flavonoids [7]. Recently, significant progress has been made in the *de novo* synthesis of polyphenolic compounds using engineered microorganisms, such as *Escherichia coli* and yeast [3].

*Cupriavidus necator* H16 (formerly *Ralstonia eutropha* H16), a well-studied H_2_-oxidizing lithoautotrophic bacterium, is capable of fixing CO_2_ via the Calvin–Benson–Bassham (CBB) cycle and storing large amounts of fixed carbon in the form of poly(3-hydroxybutyrate) (PHB) [1, 8]. Since this microbial platform is genetically tractable and has versatile metabolic capabilities, it has been engineered to produce a variety of valuable compounds from CO_2_ demonstrating its potential as an industrial workhorse [1, 9–13]. Moreover, *C. necator* H16 exhibits a faster autotrophic growth rate (doubling time = 4.2 h) than the photoautotrophic cyanobacteria (doubling time = 7–12 h) [14–16] and has been employed as a central biocatalyst in the microbial electrosynthetic systems that can use solar energy to convert CO_2_ into value added chemicals [13].

While *C. necator* H16 is considered a suitable candidate for autotrophic and electro-autotrophic chemical production, it has never been engineered for the production of polyphenolic compounds, such as resveratrol. We report here for the first time on lithoautotrophic production of resveratrol by engineered *C. necator* strains. The best-performing strains were initially screened under heterotrophic conditions, and then evaluated under lithoautotrophic conditions. An artificial phenylpropanoid pathway consisting of tyrosine ammonia lyase (*TAL*), 4-coumarate-coA ligase (*4CL*), and stilbene synthase (*STS*) was introduced into *C. necator* H16 with supplementation of _L_-tyrosine. Various metabolic strategies, including the overexpression of acetyl-CoA carboxylase (*ACC*), disruption of the PHB synthetic pathway, and an increase in the copy number of *STS* genes, were implemented to enhance resveratrol production. Finally, we produced 1.9 mg/L of resveratrol using CO_2_ as the sole carbon source via lithoautotrophic production of engineered *C. necator* strains.

## Methods

### Strains and plasmids

All strains and plasmids used in this study are listed in Table 1. *Cupriavidus necator* H16 (KCTC 22,469; Korean Collection for Type Cultures, Daejeon, South Korea) and PHB^−^4 (DSM 541, KACC 11,970; Korean Agricultural Culture Collection, Wanju, South Korea) were used as host strains for resveratrol production.

While *ACC* gene was amplified from the genomic DNA of *Corynebacterium glutamicum* ATCC 13,032, *STS*, *TAL* and *4CL* genes were codon-optimized and synthesized by IDT KOREA (Additional file: Table S2). All genes were expressed in the pBBR1-MCS2 vector under the control of the arabinose-inducible araBAD promoter. The primers used to construct the recombinant plasmids are listed in Additional file: Table S1. Plasmid preparation was performed using Mini Exprep plasmid SV (Geneall, South Korea). A QIAquick gel extraction kit (Qiagen, Germany) was used for the gel purification of DNA fragments. The restriction enzyme-based cloning method, Gibson Assembly (New England Biolabs, Massachusetts, USA), or Hifi DNA Assembly (New England Biolabs, Massachusetts, USA) was used to assemble the recombinant plasmids. To transform the constructed plasmids into *C. necator*, bacterial conjugation using *Escherichia coli* S17-1 donor strain harboring the desired plasmid was performed [17].

**Table 1:** Strains and plasmids used in this work

Strains and plasmids		Description	Sources
*Escherichia coli*			
DH10β	Used as a host for plasmid construction		Invitrogen
S17-1	Donor in conjugative plasmid transfer		
*Cupriavidus necator*			
H16	Wild type (ATCC 17,699, KCTC 22,469)		KCTC
ΔphaCAB	H16 derivative; Δ*phaCAB*		This study
PHB-4	H16 derivative; *phaC* mutation (DSM-541)		KACC
Plasmids			
pBBR1-MCS2	Broad host range plasmid; pP_lac_, Km^r^, lacZ		Addgene
pBAD	pBBR1-MCS2 derivative with araBAD promoter and araC; Km^r^		This study
pBAD-*STS-TAL-4CL*	pBAD derivative with _*Vv*_*STS*, _*Fj*_*TAL* and _*At*_*4CL*		This study
pBAD-*STS-TAL-4CL-ACC*	pBAD derivative with _*Vv*_*STS*, _*Fj*_*TAL*, _*At*_*4CL* and _*Cg*_*ACC*		This study
pBAD-*STS-TAL-4CL-ACC-STS*	pBAD derivative with 2 copies of _*Vv*_*STS*, _*Fj*_*TAL*, _*At*_*4CL* and _*Cg*_*ACC*		This study
pJQ200mp18	Suicide vector; sacB, oriV, oriT, traJ, Gm^r^		Addgene
pJQ200mp18Km	Derivative of pJQ200mp18, Km^r^		This study
pJQ200mp18Km-phaCAB	pJQ200mp18Km carrying deletion cassette for *phaCAB*		This study

### Heterotrophic and lithoautotrophic cultures

*Cupriavidus necator* H16 was routinely grown in Luria-Bertani (LB) broth at 30 ℃ and 200 rpm. For heterotrophic resveratrol fermentation, *C. necator* H16 strains were aerobically grown in 100 mL flasks containing 40 mL minimal media (MM) with 10 g/L fructose, 1.5 g/L KH_2_PO_4_, 6.74 g/L Na_2_HPO_4_•7H_2_O, 1.0 g/L (NH_4_)_2_SO_4_, 80 mg/L MgSO_4_•7H_2_O, 0.56 mg/L NiSO_4_•7H_2_O, 0.4 mg/L ferric citrate, 1 mg/L CaSO_4_•2H_2_O, and 0.5 g/L NaHCO_3_ supplemented with 5 mM tyrosine. The heterologous gene expression in the recombinant strains was induced at 6 h by the addition of 0.2% (w/v) L-arabinose unless otherwise indicated. For lithoautotrophic fermentation, the preculture was cultivated in MM with 10 g/L fructose at 30 ℃ and 200 rpm for 24 h. Cultured cells were collected by centrifugation at 4,200 rpm for 10 min, washed with MM without fructose and then inoculated into MM supplemented with 5 mM tyrosine in serum bottles. The strains were incubated at 30 ℃ and 200 rpm with the initial OD_600_ of 1 and 5, and filled with a 150 kPa of mixture gas (H_2_:O_2_:CO_2_ = 70:20:10 or 78:2:10; Airkorea Corporation, South Korea). The gas mixture was pressurized into the headspace of the 157 mL-serum bottles containing 20 mL MM every 24 h. The expression of biosynthetic genes was induced by adding 0.2% (w/v) L-arabinose after 24 h of autotrophic culture. During the cultivation of recombinant strains harboring resveratrol synthetic plasmids, kanamycin was also supplemented at a final concentration of 200 µg/mL.

### Genetic manipulation

For the preparation of the △phaCAB strain based on the sacB-knockout system, pJQ200mp18Km plasmids were constructed by amplifying homologies to the 500 bp regions upstream and downstream of the *phaCAB* operon and transferred into *C. necator* H16 via conjugation as described previously with slight modifications [15, 17]. After culture in low salt-LB broth (2.5 g/L NaCl) supplemented with 15% (w/v) sucrose and gentamicin, the deletion strains were screened by PCR with diagnostic primers (Additional file 1: Table S1).

### Analytical procedures

For the analysis of resveratrol and *p*-coumaric acid, 2 ml of the supernatant was centrifuged at 4,200 rpm for 10 min and extracted with an equal volume of ethyl acetate by vortexing. After extraction, the ethyl acetate layer was transferred to a glass tube and evaporated. The remaining residue was then dissolved in methanol. The samples filtered through a 0.2 μm syringe filter were analyzed by high-performance liquid chromatography (HPLC; Agilent technology 1100 Infinity, CA, USA) equipped with a ZORBAX SB-C18 column (4.6 × 150 mm, 3.5 μm, Agilent technology, CA, USA) maintained at 30 °C using a mobile phase composed of 40% acetonitrile and 60% water at a flow rate of 0.6 ml/min. The PHB content was quantified as previously described by Kim et al. (2022) [8].

### RNA sequencing analysis

For verification of *p*-coumaric acid utilization by *C. necator* H16, sequencing experiments were performed by E-biogen, Inc. (Seoul, South Korea). RNA-seq samples were prepared after 20 h of aerobic fermentation in a minimal medium containing 10 g/L fructose with or without 1 g/L *p*-coumaric acid. RNA-seq analysis was performed as described previously by Kim et al. (2022) [8].

## Results and discussion

### Construction of a lithoautotrophic platform for production of resveratrol from CO_2_

**Fig. 1:**
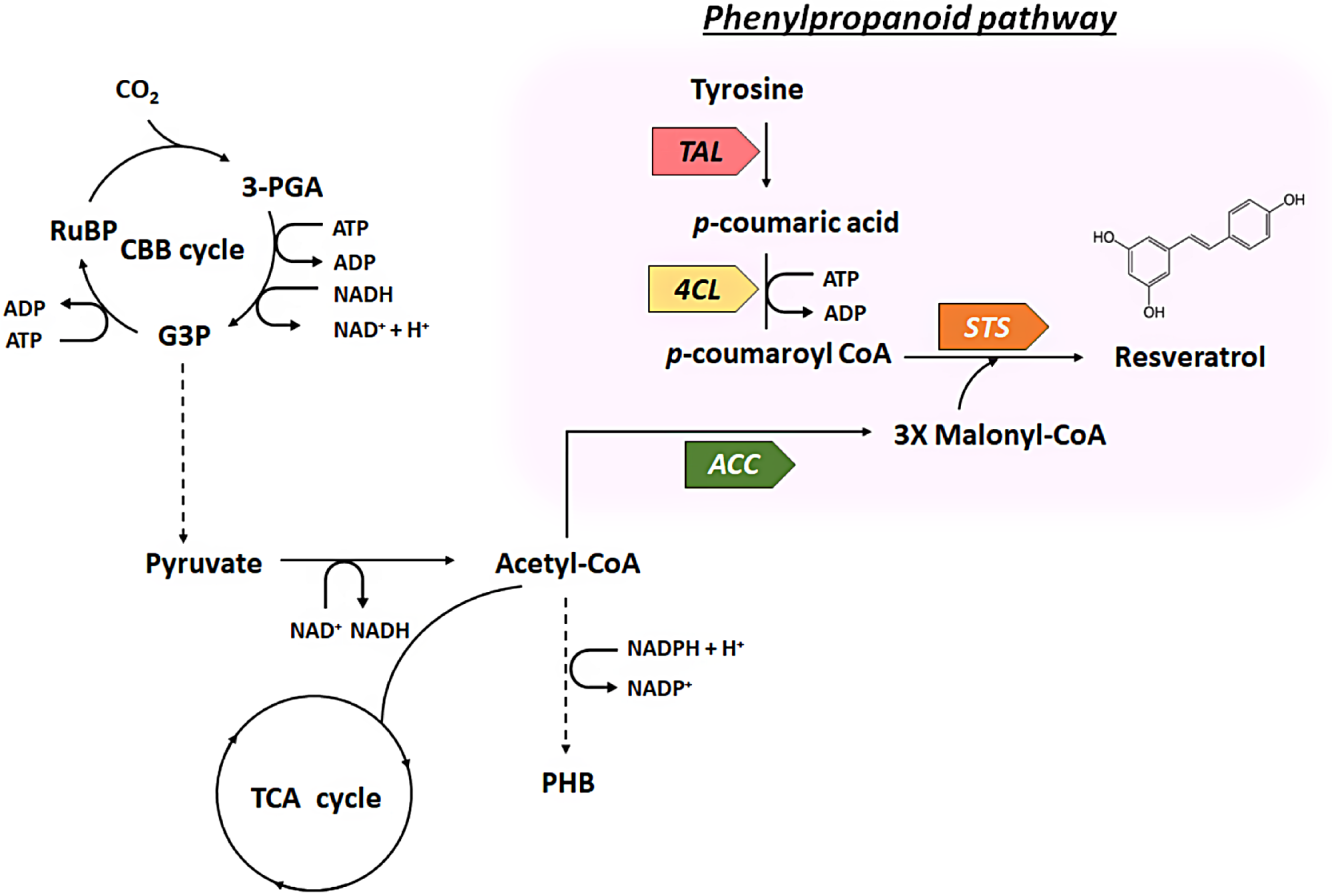
Synthetic metabolic pathways for de novo biosynthesis of resveratrol from CO_2_ and tyrosine in engineered *C. necator* H16. An artificial phenylpropanoid biosynthetic pathway engineered for resveratrol biosynthesis are shown in the colored box. The abbreviations are as follows: *TAL*, tyrosine ammonia lyase; *4CL*, 4-coumarate-CoA ligase; *STS*, stilbene synthase; *ACC*, acetyl-CoA carboxylase; 3-PGA, 3-phosphoglycerate; G3P, glyceraldehyde-3-phosphate; RuBP, ribulose biphosphate. The dashed lines indicates omitted reaction steps

A lithoautotrophic microbial platform was designed to produce resveratrol from CO_2_ via a phenylpropanoid biosynthetic pathway (Fig. 1). As the starting point, we expressed tyrosine ammonia lyase (*TAL*), which converts L-tyrosine to *p*-coumaric acid. In previous studies, microbial production of resveratrol was achieved with the supplementation of the precursor *p*-coumaric acid or aromatic amino acids such as L-tyrosine [2, 18]. The aromatic amino acid tyrosine is formed via the shikimate pathway in plants and microorganisms [19]. In this study, 5 mM tyrosine was used as the starting precursor for resveratrol synthesis. Consequently, *p*-coumaroyl-CoA is formed from the conversion of *p*-coumaric acid by expressing 4-coumarate: coenzyme ligase (*4CL*). Stilbene synthase (*STS*) sequentially directs the condensation of one molecule of *p*-coumaroyl-CoA with three malonyl-CoA molecules to produce resveratrol [18] (Fig. 1).

**Fig. 2:**
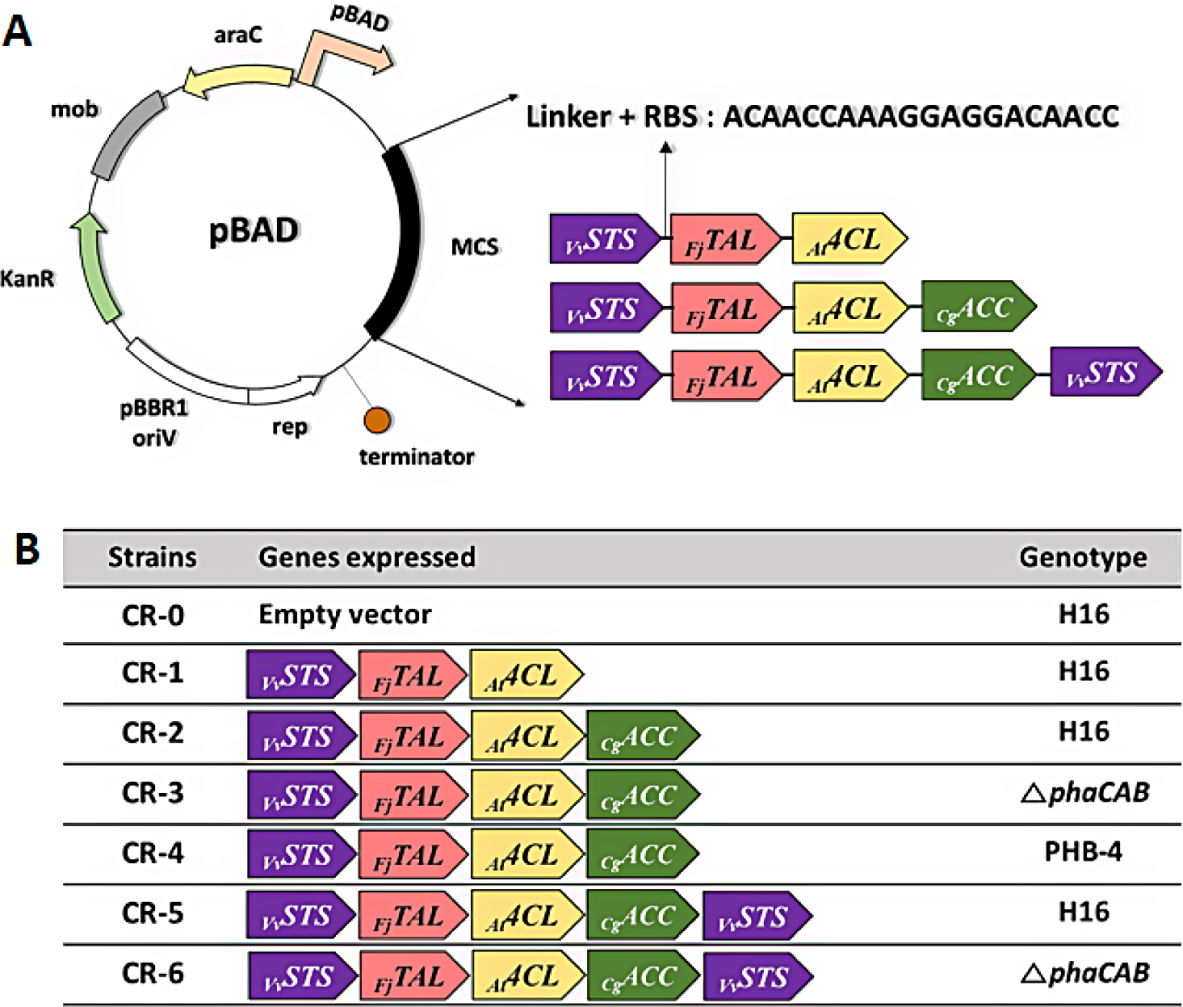
Construction of recombinant *C. necator* strains. (**A**) Genetic map of a resveratrol biosynthetic plasmid containing a synthetic ribosome-binding site (RBS) and a nucleotide linker sequence inserted between each gene [20]. (**B**) List of engineered strains with different combinations of heterologous genes. The sources of heterologous genes are indicated: _*Vv*_*STS, Vitis vinifera STS*; _*Fj*_*TAL*, *Flavobacterium johnsoniae*; _*At*_*4CL, Arabidopsis thaliana 4CL*; _*Cg*_*ACC, Corynebacterium glutamicum ACC*

To achieve resveratrol production from CO_2_, we constructed three recombinant plasmids carrying an *STS* gene from *Vitis vinifera* (_*Vv*_*STS*), a *TAL* gene from *Flavobacterium johnsoniae* (_*Fj*_*TAL*), a *4CL* gene from *Arabidopsis thaliana* (_*At*_*4CL*) and a *ACC* gene from *Corynebacterium glutamicum* (_*Cg*_*ACC*) under the control of arabinose-inducible araBAD promoter (Fig. 2A). To enhance the carbon flux of *p*-coumaroyl-CoA towards the resveratrol biosynthetic pathway, the recombinant plasmid containing a second copy of _*Vv*_*STS* was also constructed. Since the distance from the promoter affects the gene expression level [20], another _*Vv*_*STS* gene was placed in the last order to ensure the strong expression of _*Fj*_*TAL*, _*At*_*4CL* and _*Cg*_*ACC*. The recombinant strains tested in this study are shown in Fig. 2B. First, the cell growth of *C. necator* H16 in the presence of resveratrol ranging from 0 to 50 mg/L was tested to evaluate the potential problems with the toxicity of resveratrol to cells (Additional file 1: Fig. S1). Notably, the presence of 50 mg/L resveratrol caused severe retardation of *C. necator* H16 cell growth.

### Evaluation of heterotrophic resveratrol production by engineered strains

**Fig. 3:**
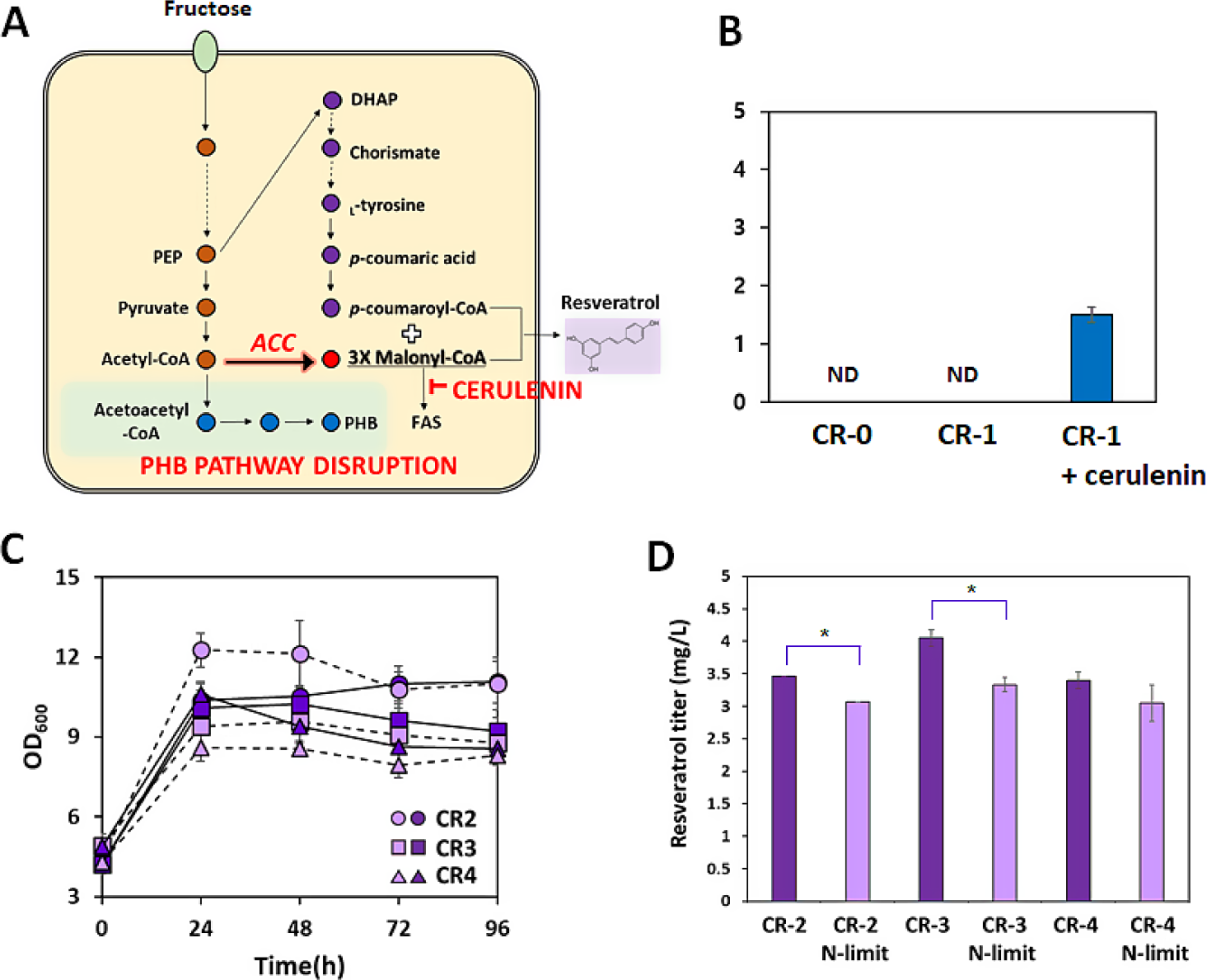
Resveratrol production using engineered strains under heterotrophic conditions. (**A**) Strategies for increased malonyl-CoA availability and resveratrol synthesis. The strategies used in this study including the overexpression of *ACC*, addition of cerulenin and disruption of PHB synthetic pathway are stated as red colors. The dashed lines indicates omitted reaction steps. The abbreviations are as follows: *ACC*, acetyl-CoA carboxylase; PEP, phosphoenolpyruvate; DHAP, 3-deoxy-D-arabinoheptulosanate-7-phosphate; FAS, fatty acid synthesis. (**B**) Resveratrol production using the CR-1 strain with or without cerulenin supplementation. (**C**) Cell growth and (**D**) resveratrol production using the CR-2, CR-3, and CR-4 strains. Cells were grown in either nitrogen-rich MM medium (solid lines, dark purple) containing 10 g/L fructose and 1 g/L (NH_4_)_2_SO_4_ or nitrogen-limiting MM (dashed lines, light purple) containing 10 g/L fructose and 0.5 g/L (NH_4_)_2_SO_4_. The MM medium was supplemented with 5 mM tyrosine for resveratrol synthesis. The resveratrol titer was measured at the end of 96 h-fermentation. Error bars represent the standard deviation from three biological replicates. Student’s two-tailed t-test was performed to determine the significance of differences (*, *p* < 0.05)

The engineered strains were evaluated for their resveratrol production capabilities, using fructose as the carbon source (Fig. 3). When the CR-1 strain carrying _*Fj*_*TAL*, _*At*_*4CL*, and _*Vv*_*STS* genes was cultured in a minimal medium supplemented with 5 mM tyrosine, resveratrol synthesis was not detected (Fig. 3B). Since the biosynthesis of resveratrol requires three moles of malonyl-CoA, limited malonyl-CoA availability may be the decisive bottleneck in the native metabolism of *C. necator* H16. Most microorganisms generate malonyl-CoA solely from irreversible acetyl-CoA carboxylation catalyzed by acetyl-CoA carboxylases (ACCs), which is a key precursor of *de novo* fatty acid synthesis (FAS) [21]. Since the malonyl-CoA pool is highly controlled and consumed by FAS [21, 22], FAS is regarded as an undesired metabolic pathway in terms of resveratrol biosynthesis [22]. Therefore, we used two strategies to increase intracellular malonyl-CoA availability: inhibition of fatty acid synthesis and overexpression of acetyl-CoA carboxylase (*ACC*) (Fig. 3A).

To inhibit the synthesis of fatty acids that incorporate malonyl-CoA, 0.05 mM of the FAS inhibitor cerulenin was supplemented into the medium (Fig. 3B). The CR-1 strain supplemented with cerulenin produced 1.5 mg/L of resveratrol. This indicates that the limited availability of malonyl-CoA is a major bottleneck in resveratrol synthesis in *C. necator* H16. Cerulenin supplementation has been shown to enhance polyphenolic compound production in *E. coli* and *C. glutamicum* in prior studies [7, 23, 24]. However, it is expensive and can inhibit cell growth due to irreversible and non-selective FAS inhibition [22, 25–27]. To address this, gene downregulation strategies using antisense RNA or CRISPR interference have been used in previous studies for malonyl-CoA-derived compounds [28, 29].

Instead of reducing undesired malonyl-CoA consumption by adding cerulenin, we constructed a CR-2 strain overexpressing *ACC* derived from *C. glutamicum* to increase the intracellular malonyl-CoA pool. Since *ACC* of *C. glutamicum* comprises only two subunits (*accBC* and *dtsR1*) instead of a four-subunit protein complex of *ACC* derived from *E. coli* and others for catalytic activity [30], this enzyme was overexpressed in *C. necator* H16. The expression of heterologous *ACC* genes in *C. necator* H16 resulted in successful resveratrol synthesis, producing 3.5 mg/L (Fig. 3D).

To further channel more carbon flux from acetyl-CoA towards malonyl-CoA, we disrupted the PHB synthesis pathway. Using the sacB-based gene knock-out plasmid, the PHB non-producing strain ΔphaCAB was obtained with the deletion of the entire *phaCAB* operon encoding the essential enzymes for PHB synthesis (Table 1). We also employed a representative PHB-negative mutant, PHB^−^4 (DSM 541), which has a single nonsense mutation in the PHA synthase gene *phaC* [31]. Although PHB accumulation was not detected, both the PHB-negative PHB^−^4 and △CAB strains showed retarded cell growth in media containing fructose (10 g/L) as the carbon source (Additional file 1: Fig. S2). Since acetyl-CoA is not consumed in the PHB synthetic pathway, mutant cells might have altered cellular metabolism with the accumulation of metabolites, such as acetyl-CoA and pyruvate [31]. When *C. necator* H16, △CAB, and PHB^−^4 strains carried the pBAD-*STS-TAL-4CL-ACC* plasmid, the resulting strains CR-2, CR-3, and CR-4 produced 3.5, 4.1, and 3.5 mg/L of resveratrol, respectively (Fig. 3D). Although the cell growth rate of CR-3 was lower than that of CR-2, its resveratrol titer was obtained as 4.1 mg/L, 17% higher than that of CR-2. Disruption of the competing pathway by deleting the entire *phaCAB* operon effectively enhanced resveratrol synthesis. However, resveratrol production by the PHB^−^4 strain (CR-4) did not show any positive result and its growth rate was lower than that of CR-3. Therefore, the CR-3 strain with the highest resveratrol synthesis capability was selected for further engineering. Since cell growth and PHB synthesis in *C. necator* H16 depend on the nitrogen concentration with excess carbon available, the effect of nitrogen supplementation on resveratrol production was investigated. Also, the resveratrol production performances of the different strains with or without the PHB synthetic pathway were compared. While nitrogen limitation generally boosts PHB accumulation [32], the resveratrol synthesis was negatively affected under the nitrogen limitation condition (0.5 (NH_4_)_2_SO_4_-g/L). Although the CR-2 strain showed the enhanced cell growth at 24 h under nitrogen limitation (Fig. 3C), it led to the decreased resveratrol production. For the PHB-negative CR-3 and CR-4 strains, nitrogen limitation led to decreased cell growth, resulting in reduced resveratrol production (Fig. 3C, D). When the competing pathway was disrupted to avoid the depletion of acetyl-CoA, which is the precursor to PHB, in CR-3 and CR-4 strains, the redirected carbon flow towards acetyl-CoA under nitrogen limitation could not increase resveratrol synthesis. While the sufficient nitrogen supply is required for enhancing cell growth and resveratrol production, the > 2-fold enhancement of 1,3-butanediol production by engineered *C. necator* H16 was observed under nutrient-limiting conditions [15]. Despite decreased cell growth, the availability of 3-hydroxybutyryl-CoA precursor boosted by nitrogen limitation enabled the enhanced 1,3-butanediol production [15].

### Additional *STS* copy increases the flux of *p*-coumaroyl CoA towards resveratrol biosynthesis

**Fig. 4:**
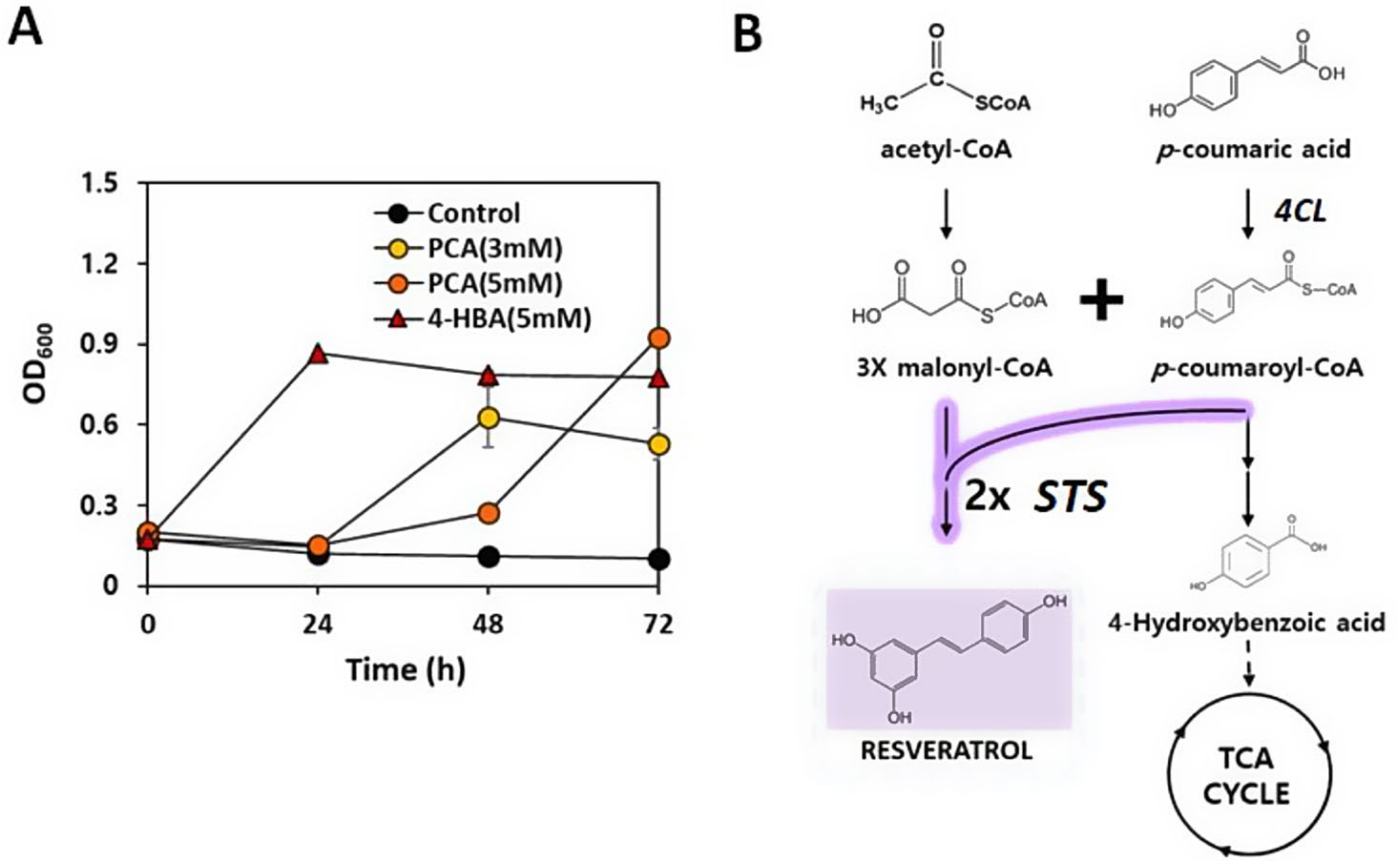
Coumarate catabolic pathways in *C. necator* H16 causes the loss of *p*-coumaroyl CoA, an important precursor for resveratrol synthesis. (**A**) Cell growth of *C. necator* H16 strain on various aromatic compounds as the sole carbon source. PCA: *p*-coumaric acid, 4-HBA: 4-hydroxybenzoic acid. The initial optical density at 600 nm (OD_600_) was 0.2 at 600 nm and the initial *p*-coumaric acid and 4-hydroxybenzoic acid concentrations were 3 and 5 mM, respectively. (**B**) Metabolic engineering of *C. necator* H16 for re-directing malonyl-CoA flux towards resveratrol synthesis by addition of _*Vv*_*STS*

**Fig. 5:**
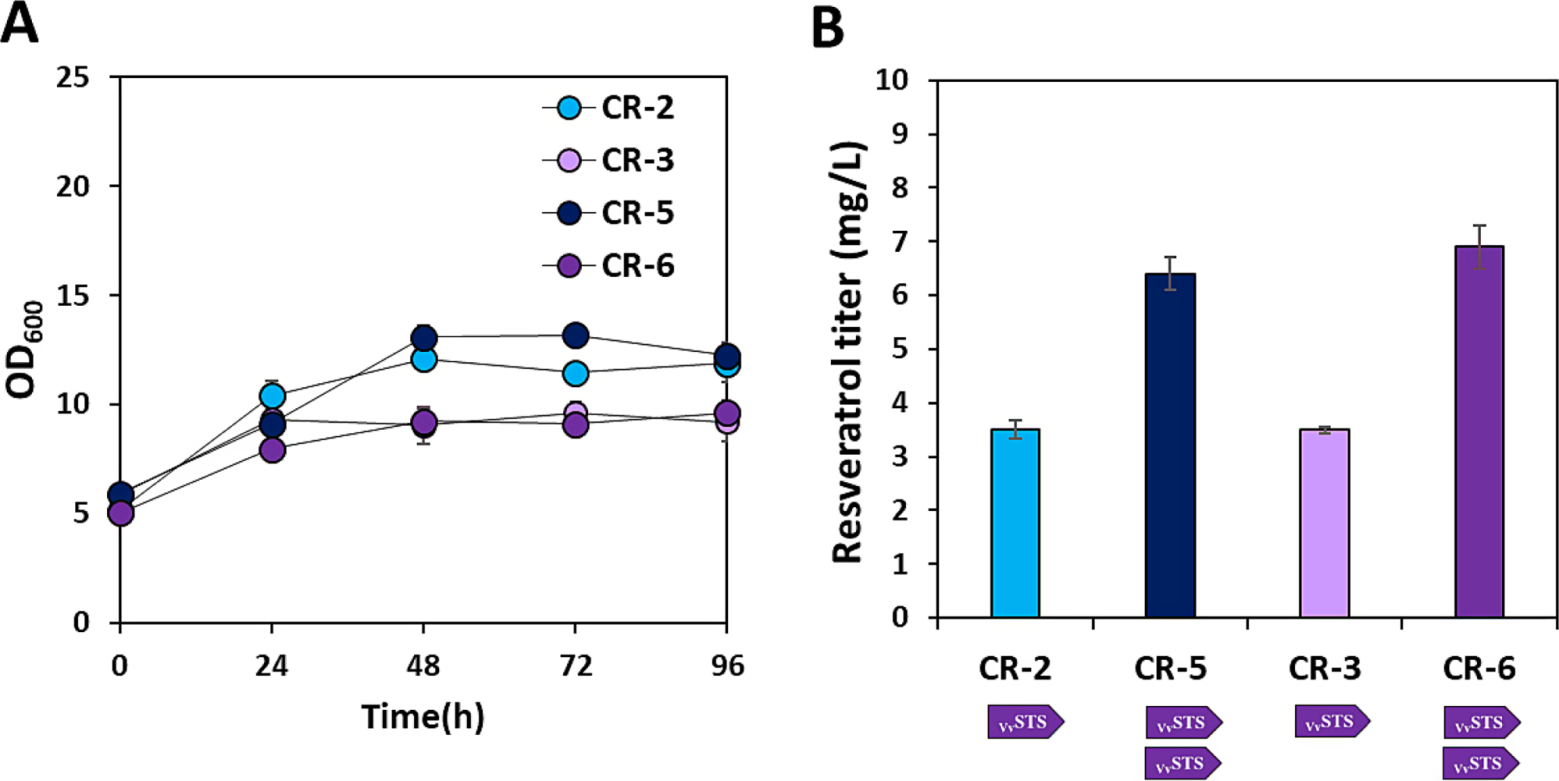
Effects of increasing the copy number of _*Vv*_*STS* and disrupting the PHB pathway on resveratrol production under heterotrophic conditions. (**A**) Cell growth curve and (**B**) resveratrol production in the CR-2, CR-3, CR-5, and CR-6 strains. The initial OD_600_ was 5 and the initial fructose concentration was 10 g/L. The MM medium was supplemented with 5 mM tyrosine for resveratrol synthesis. The resveratrol titer was measured at the end of 96 h-fermentation

While malonyl-CoA availability was regulated by overexpression of *ACC* and disruption of the PHB synthetic pathway, yielding 4.1 mg/L of resveratrol, one molecule of *p*-coumaroyl-CoA via coumaric acid conversion by the action of *4CL* was still required for resveratrol synthesis. Although high levels of *p*-coumaric acid are toxic to cells, the possibility of coumaric acid as a carbon source of *C. necator* H16 at low concentrations has been suggested in previous studies [33]. The catabolic mechanisms of lignin-derived aromatic compounds, including coumaric, ferulic, and cinnamic acids, have not been completely elucidated in *C. necator* H16. It has been suggested that *p*-coumarate is converted to 4-hydroxybenzoate and then fed into the TCA cycle [33]. As shown in Fig. 4A, the growth of *C. necator* H16 was observed in the presence of 3–5 mM *p*-coumaric acid as the sole carbon source, although a high concentration of *p*-coumaric acid (5 mM) led to a prolonged lag phase. Although the genes responsible for the conversion of *p*-coumaric acid to 4-hydroxybenzoate were not completely identified, cell growth on 4-hydroxybenzoate and the upregulation of the genes involved in the reactions starting from 4-hydroxybenzoate to succinyl-CoA were observed when the cells were cultured with supplementation of *p-*coumaric acid (Additional file 1: Fig. S3). This indicates that tyrosine supplemented in the medium as the precursor for resveratrol synthesis might be converted to primary metabolites in the engineered strains. As the activation of coumarate catabolic pathways in *C. necator* H16 causes the loss of *p*-coumaroyl CoA, an important precursor for resveratrol synthesis, we aimed to redirect the carbon flux of *p*-coumaroyl-CoA towards the resveratrol biosynthetic pathway by overexpressing two copies of _*Vv*_*STS* (Fig. 4B). The addition of a second copy of _*Vv*_*STS* in the CR-2 and CR-3 strains, resulting in CR-5 and CR-6 strains, increased resveratrol production from 3.5 to 4.1 mg/L to 6.4 and 6.9 mg/L, respectively (Fig. 5B). The final OD_600_ values at 96 h of CR-5 and CR-6 strains were not significantly different from those of CR-2 and CR-3 strains, respectively (Fig. 5A). The > 70% increase in resveratrol synthesis in the CR-5 and CR-6 strains demonstrated that increasing STS activity is important to make use of the available *p*-coumaroyl-CoA. With further efforts to understand the metabolism of aromatic compounds in *C. necator* H16, the identification, and deletion of key genes involved in the conversion of *p*-coumaroyl-CoA to 4-hydroxybenzoate could be an alternative strategy for increasing *p*-coumaroyl-CoA availability for resveratrol production. Incha et al. (2020) identified genes involved in the coumarate catabolic pathways of *Pseudomonas putida* KT2440 that could potentially impact *p*-coumaroyl-CoA-derived products. In engineered *P. putida*, deletion of the gene *ech* (enoyl-CoA hydrolase-lyase) in coumarate catabolism increased type III polyketide bisdemethoxycurcumin [34].

Microbial production of resveratrol has been reported in various microorganisms (Table 2). In particular, significant efforts in metabolic engineering toward high-level resveratrol production have been achieved in *E. coli*, *C. glutamicum*, *Saccharomyces cerevisiae*, and *Yarrowia lipolytica* [3]. While the first production of resveratrol with the titer of 1.45 µg/L was achieved in engineered yeast [35], a high level of resveratrol production reaching approximately 800 mg/L was found in engineered *S. cerevisiae* by optimized fed-batch fermentation [36]. With advancements in synthetic biology tools, there have been numerous efforts to regulate metabolic pathways and optimize resveratrol production; *E. coli* and *C. glutamicum* have yielded 300 and 110 mg/L of resveratrol titer, respectively [7, 18, 25, 36, 37]. Other bacterial hosts, such as *Streptomyces venezuelae* and *Aspergillus niger* have also been used to produce resveratrol by introducing a heterologous phenylpropanoid biosynthetic pathway [38, 39]. It was reported that engineered *S. venezuela* produced resveratrol for the first time, with a yield of just 0.4 mg/L [39]. In this study, we achieved 6.8 mg/L of resveratrol from fructose in engineered *C. necator* H16, which is the first time that a polyphenolic compound has been synthesized in this host. Taken together, the CR-6 strain constructed in this study represents a promising starting point for further engineering towards a more efficient resveratrol production.

**Table 2:** Heterotrophic and autotrophic microbial production of resveratrol in various engineered strains

Host strain	Characteristics	Carbon source	Titer (mg/L)	Reference
*C. necator* H16	2x*STS*, *TAL, 4CL*	Fructose	6.8	This study
	2x*STS*, *TAL, 4CL*	CO_2_	1.9	This study
*S. elogatus*	*AROG, SAM8, 4CL, STS*	CO_2_	4.6	[40]
*L. lactis*	*TAL,4CL, STS, ACC*	Glucose	1.3	[41]
*S. venezuelae*	*STS,4CL*, Pikromycin *pks* deletion	Sucrose	0.4	[39]
*S. cerevisiae*	*PAL, 4CL, STS, ACC1*	Galactose	5.8	[42]
*S. cerevisiae*	*PAL, C4H, 4CL, VST, ACS, ATR2, ARO4*^*fbr*^, *ARO7*^*fbr*^, *CYB5, ACC1, △ARO10*	Glucose	812	[36]
*Y. lipolytica*	*4CL, STS, PEX10, ACC1*	Glucose	48.7	[43]
*E. coli*	*PAL, 4CL, STS, ACC*	Glucose	37	[44]
*E. coli*	*TAL, 4CL, STS, ACC, GroEL, GroES, ompF*, anti-*fabD*	Glycerol	238	[18]

### Lithoautotrophic production of resveratrol from CO_2_

**Fig. 6:**
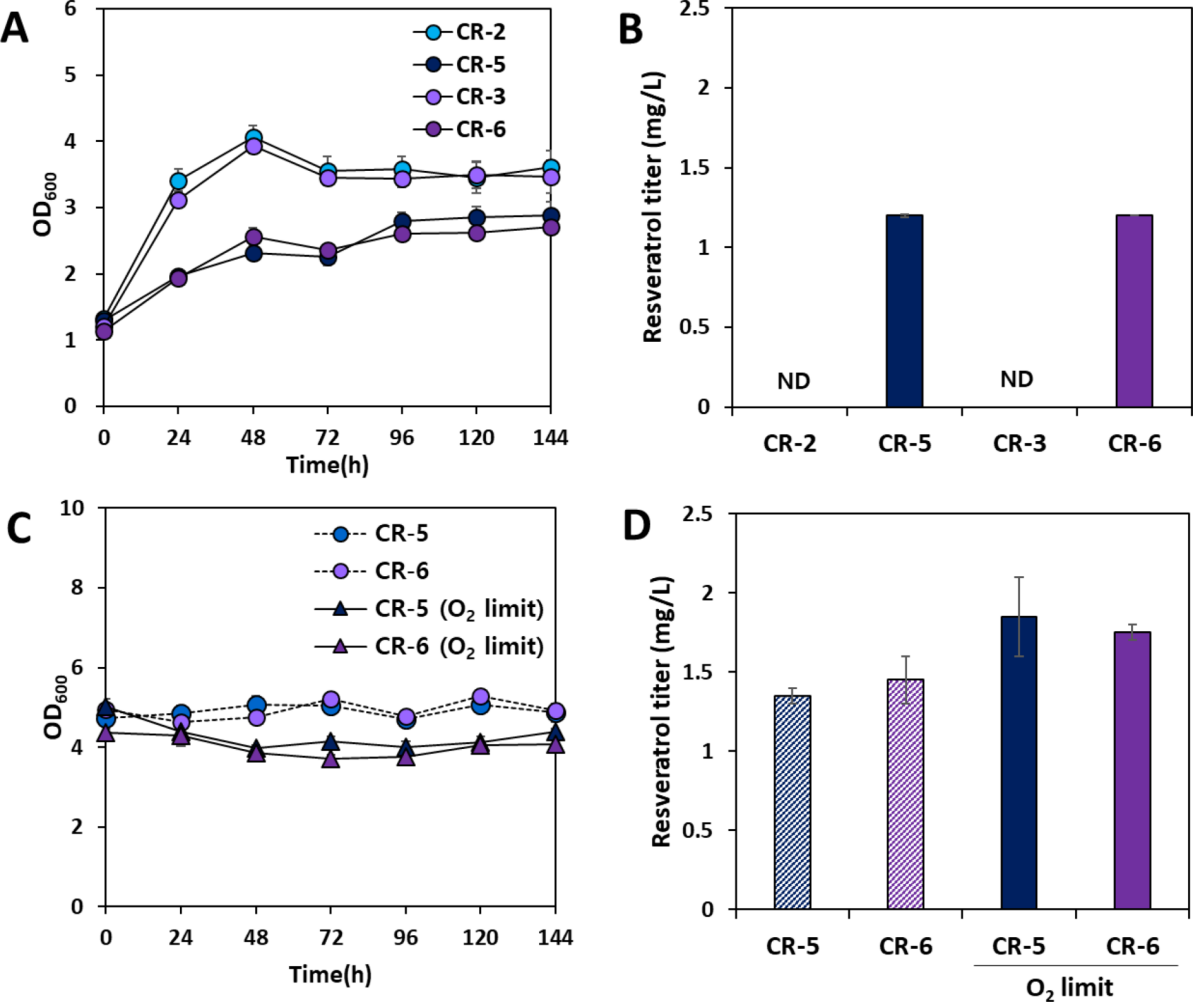
Resveratrol production using engineered strains from CO_2_ and tyrosine. Lithoautotrophic fermentation profiles of the engineered strains for resveratrol production under different gas compositions of (**A**, **B**) H_2_:O_2_:CO_2_ = 70:20:10 vs. (**C**, **D**) H_2_:O_2_:CO_2_ = 88:2:10 (O_2_-limited) with different initial cell densities. The MM medium was supplemented with 5 mM tyrosine for resveratrol synthesis and the expression of resveratrol-biosynthetic genes was induced by adding 0.2% (w/v) L-arabinose after 24 h of autotrophic culture. The resveratrol titer was measured at the end of 144 h-fermentation

The final goal of this study was to produce resveratrol from CO_2_. For autotrophic production of resveratrol, the recombinant strains CR-2, CR-3, CR-5, and CR-6 were cultivated in serum bottles supplemented with CO_2_, H_2_, and O_2_ (Fig. 6). Although the cell growth rates of the CR-2 and CR-3 strains were significantly higher than those of the CR-5 and CR-6 strains with initial cell densities (OD_600_) of 1 (Fig. 6A), resveratrol synthesis was not observed in the CR-2 and CR-3 strains (Fig. 6B). Approximately 1.2 mg/L of resveratrol was produced from CO_2_ and tyrosine by the CR-5 and CR-6 strains carrying a second copy of _*Vv*_*STS*. However, differences in autotrophic performance between the CR-5 and CR-6 strains were not observed (Fig. 6B). Since the high cell density culture offers an efficient way to enhance the microbial fermentation productivities [45], the autotrophic cultures with the higher initial cell densities were performed. When the initial cell density was increased from 1 to 5, both the CR-5 and CR-6 strains produced slightly more resveratrol, yielding 1.4 and 1.5 mg/L, respectively (Fig. 6C and D). In addition, autotrophic fermentation under oxygen stress (H_2_:O_2_:CO_2_ = 88:2:10) was performed to promote resveratrol synthesis. Although *C. necator* H16 is typically cultured in a gas composition of H_2_:O_2_:CO_2_ = 70:20:10 for autotrophic growth, some previous works have reported that oxygen stress (< 3% O_2_) induced enhanced PHB production while restricting cell growth [46]. When oxygen stress (H_2_:O_2_:CO_2_ = 88:2:10) was induced in the CR-5 and CR-6 strains, resveratrol production increased from 1.4 to 1.5 mg/L to 1.8–1.9 mg/L.

Although *C. necator* H16 has been used as a host for the potential commercial production of polyhydroxyalkanoates (PHAs), it has been engineered to produce a variety of chemicals, such as isopropanol, acetoin, humulene, methyl ketone, *(R)*-1,3-butanediol, and alkene, under both heterotrophic and autotrophic conditions (Table 3) [1, 9, 10, 12, 15]. More recently, the CO_2_ conversion into sugars and value-added compounds by engineered *C. necator* strains using H_2_ as an energy source produced 470 mg/L trehalose, 250 mg/L glucose, and 1.7 mg/L lycopene [11, 13, 47]. This work is the first step towards the production of plant type III PKS-derived compounds in *C. necator*, but the synergistic combination of metabolic and fermentation optimization strategies for increasing productivity should be addressed in the future. With further studies on improving efficiencies of carbon fixation and resveratrol synthesis, a better understanding of carbon flux of CO_2_ in resveratrol-producing *C. necator* strains should allow us to enhance the viability of lithoautotrophic biochemical production. Due to the inhibitory effect of resveratrol on the cell growth of *C. necator* H16 (Additional file: Fig. S1), further metabolic engineering strategies for enhancing microbial tolerance towards resveratrol as well as the application of in situ product removal strategies are required to avoid the product toxicity limitations. Since the engineered *C. necator* strains still require the supplementation of exogenous tyrosine as a precursor, the metabolic engineering efforts on *de novo* tyrosine biosynthetic pathway is also needed to produce resveratrol solely from CO_2_.

**Table 3:** Valorization of CO_2_ into valuable chemicals by engineered *C. necator* H16

Target product	Pathway used	Titer (mg/L)	Reference
Resveratrol	Phenylpropanoid pathway	1.9	This study
Lycopene	Isopentenyl pyrophosphate (IPP) and dimethylallyl diphosphate (DMAPP)-dependent pathway	1.7	[13]
1,3-butanediol	3-hydroxybutyraldehyde-CoA-(3HB-CoA) and pyruvate-dependent pathway	2,970	[15]
Isopropanol	Acetoacetyl-CoA based pathway	250	[1]
Humulene*	Mevalonate pathway and humulene synthase	10.8	[10]
Methyl ketone	TesA thioesterase dependent pathway	50–180	[12]
Alkene	Acyl-ACP reductase (AAR) and alde- hyde deformylating oxygenase (ADO)	4.4	[9]
Trehalose	Expression of transport proteins for secretion	470	[11]
Glucose	Blocking the glucose catabolic pathway via deletion of *glk*	250	[47]

*Chemicals produced electroautotrophically via microbial electrosynthesis (MES)

## Conclusions

In this study, a microbial lithoautotrophic platform was developed by introducing an artificial resveratrol biosynthetic pathway into *C. necator* H16. Implementation of phenylpropanoid pathways consisting of tyrosine ammonia lyase (*TAL*), 4-coumarate-coA ligase (*4CL*), and stilbene synthase (*STS*) with supplementation of _L_-tyrosine in combination with disrupting PHB biosynthesis and enhancing carbon flux by increasing copies of *STS* enabled the strain to produce 1.9 mg/L of resveratrol from CO_2_ and tyrosine. Further intensive efforts on the metabolic engineering of phenylpropanoid and tyrosine-biosynthetic pathways will be necessary to realize the industrial production of resveratrol from CO_2_. The recombinant *C. necator* strain developed in this study is considered a promising starting strain for further engineering to produce polyphenolic compounds. With the continued engineering of *C. necator* H16, this strain will become a more attractive lithoautotrophic platform, providing a sustainable route for the valorization of CO_2_ to high-value natural products.

### Electronic supplementary material

Below is the link to the electronic supplementary material.

Supplementary Material 1

## Data Availability

All the data for this study are available within this published article and its additional files.

## References

[1] Marc J, Grousseau E, Lombard E, Sinskey AJ, Gorret N, Guillouet SE (2017). Over expression of GroESL in *Cupriavidus necator* for heterotrophic and autotrophic isopropanol production. Metab Eng.

[2] Noda S, Kondo A (2017). Recent advances in microbial production of aromatic chemicals and derivatives. Trends Biotechnol.

[3] Feng C, Chen J, Ye W, Liao K, Wang Z, Song X, Qiao M (2022). Synthetic biology-driven microbial production of resveratrol: advances and perspectives. Front Bioeng Biotechnol.

[4] Lu Y, Shao D, Shi J, Huang Q, Yang H, Jin M (2016). Strategies for enhancing resveratrol production and the expression of pathway enzymes. Appl Microbiol Biotechnol.

[5] Thapa SB, Pandey RP, Park YI, Kyung Sohng J. Biotechnological advances in resveratrol production and its chemical diversity. Molecules. 2019;24. 10.3390/molecules24142571 PMC6680439 31311182

[6] Sáez-Sáez J, Wang G, Marella ER, Sudarsan S, Cernuda Pastor M, Borodina I (2020). Engineering the oleaginous yeast *Yarrowia Lipolytica* for high-level resveratrol production. Metab Eng.

[7] Lim CG, Fowler ZL, Hueller T, Schaffer S, Koffas MA (2011). High-yield resveratrol production in engineered *Escherichia coli*. Appl Environ Microbiol.

[8] Kim S, Jang YJ, Gong G, Lee S-M, Um Y, Kim KH, Ko JK (2022). Engineering *Cupriavidus necator* H16 for enhanced lithoautotrophic poly(3-hydroxybutyrate) production from CO_2_. Microb Cell Fact.

[9] Crépin L, Lombard E, Guillouet SE (2016). Metabolic engineering of *Cupriavidus necator* for heterotrophic and autotrophic alka(e)ne production. Metab Eng.

[10] Krieg T, Sydow A, Faust S, Huth I, Holtmann D (2018). CO_2_ to terpenes: autotrophic and electroautotrophic α-humulene production with *Cupriavidus necator*. Angew Chem Int Ed Engl.

[11] Löwe H, Beentjes M, Pflüger-Grau K, Kremling A (2021). Trehalose production by *Cupriavidus necator* from CO_2_ and hydrogen gas. Bioresour Technol.

[12] Müller J, MacEachran D, Burd H, Sathitsuksanoh N, Bi C, Yeh YC, Lee TS, Hillson NJ, Chhabra SR, Singer SW, Beller HR (2013). Engineering of *Ralstonia eutropha* H16 for autotrophic and heterotrophic production of methyl ketones. Appl Environ Microbiol.

[13] Wu H, Pan H, Li Z, Liu T, Liu F, Xiu S, Wang J, Wang H, Hou Y, Yang B, Lei L, Lian J (2022). Efficient production of lycopene from CO_2_ via microbial electrosynthesis. Chem Eng J.

[14] Bernstein HC, McClure RS, Hill EA, Markillie LM, Chrisler WB, Romine MF, McDermott JE, Posewitz MC, Bryant DA, Konopka AE, Fredrickson JK, Beliaev AS. Unlocking the constraints of cyanobacterial productivity: acclimations enabling ultrafast growth. mBio. 2016;7. 10.1128/mbio.00949. 10.1128/mBio.00949-16 PMC4981716 27460798

[15] Gascoyne JL, Bommareddy RR, Heeb S, Malys N (2021). Engineering *Cupriavidus necator* H16 for the autotrophic production of (R)-1,3-butanediol. Metab Eng.

[16] Lütte S, Pohlmann A, Zaychikov E, Schwartz E, Becher JR, Heumann H, Friedrich B (2012). Autotrophic production of stable-isotope-labeled arginine in *Ralstonia eutropha* strain H16. Appl Environ Microbiol.

[17] Xiong B, Li Z, Liu L, Zhao D, Zhang X, Bi C (2018). Genome editing of *Ralstonia eutropha* using an electroporation-based CRISPR-Cas9 technique. Biotechnol Biofuels.

[18] Zhao Y, Wu BH, Liu ZN, Qiao J, Zhao GR (2018). Combinatorial optimization of resveratrol production in engineered *E. Coli*. J Agric Food Chem.

[19] Averesch NJH, Krömer JO (2018). Metabolic engineering of the shikimate pathway for production of aromatics and derived compounds-present and future strain construction strategies. Front Bioeng Biotechnol.

[20] Grousseau E, Lu J, Gorret N, Guillouet SE, Sinskey AJ (2014). Isopropanol production with engineered *Cupriavidus necator* as bioproduction platform. Appl Microbiol Biotechnol.

[21] Zha W, Rubin-Pitel SB, Shao Z, Zhao H (2009). Improving cellular malonyl-CoA level in *Escherichia coli* via metabolic engineering. Metab Eng.

[22] Milke L, Marienhagen J (2020). Engineering intracellular malonyl-CoA availability in microbial hosts and its impact on polyketide and fatty acid synthesis. Appl Microbiol Biotechnol.

[23] Leonard E, Lim KH, Saw PN, Koffas MA (2007). Engineering central metabolic pathways for high-level flavonoid production in *Escherichia coli*. Appl Environ Microbiol.

[24] Kallscheuer N, Vogt M, Stenzel A, Gätgens J, Bott M, Marienhagen J (2016). Construction of a *Corynebacterium glutamicum* platform strain for the production of stilbenes and (2S)-flavanones. Metab Eng.

[25] Milke L, Ferreira P, Kallscheuer N, Braga A, Vogt M, Kappelmann J, Oliveira J, Silva AR, Rocha I, Bott M, Noack S, Faria N, Marienhagen J (2019). Modulation of the central carbon metabolism of *Corynebacterium glutamicum* improves malonyl-CoA availability and increases plant polyphenol synthesis. Biotechnol Bioeng.

[26] Price AC, Choi K-H, Heath RJ, Li Z, White SW, Rock CO (2001). Inhibition of β-ketoacyl-acyl carrier protein synthases by thiolactomycin and cerulenin: structure and mechanism. J Biol Chem.

[27] Giner-Robles L, Lázaro B, de la Cruz F, Moncalián G (2018). fabH deletion increases DHA production in *Escherichia coli* expressing pfa genes. Microb Cell Fact.

[28] Wu J, Du G, Chen J, Zhou J (2015). Enhancing flavonoid production by systematically tuning the central metabolic pathways based on a CRISPR interference system in *Escherichia coli*. Sci Rep.

[29] Yang Y, Lin Y, Li L, Linhardt RJ, Yan Y (2015). Regulating malonyl-CoA metabolism via synthetic antisense RNAs for enhanced biosynthesis of natural products. Metab Eng.

[30] Wang S, Jin X, Jiang W, Wang Q, Qi Q, Liang Q (2022). The expression modulation of the key enzyme Acc for highly efficient 3-hydroxypropionic acid production. Front Microbiol.

[31] Raberg M, Voigt B, Hecker M, Steinbüchel A (2014). A closer look on the polyhydroxybutyrate- (PHB-) negative phenotype of *Ralstonia eutropha* PHB-4. PLoS ONE.

[32] Tian J, Sinskey AJ, Stubbe J (2005). Kinetic studies of polyhydroxybutyrate granule formation in *Wautersia eutropha* H16 by transmission electron microscopy. J Bacteriol.

[33] Wang W, Yang S, Hunsinger GB, Pienkos PT, Johnson DK (2014). Connecting lignin-degradation pathway with pre-treatment inhibitor sensitivity of *Cupriavidus necator*. Front Microbiol.

[34] Incha MR, Thompson MG, Blake-Hedges JM, Liu Y, Pearson AN, Schmidt M, Gin JW, Petzold CJ, Deutschbauer AM, Keasling JD (2020). Leveraging host metabolism for bisdemethoxycurcumin production in *Pseudomonas putida*. Metab Eng Commun.

[35] Becker JV, Armstrong GO, van der Merwe MJ, Lambrechts MG, Vivier MA, Pretorius IS (2003). Metabolic engineering of *Saccharomyces cerevisiae* for the synthesis of the wine-related antioxidant resveratrol. FEMS Yeast Res.

[36] Li M, Schneider K, Kristensen M, Borodina I, Nielsen J (2016). Engineering yeast for high-level production of stilbenoid antioxidants. Sci Rep.

[37] Wu J, Zhou P, Zhang X, Dong M (2017). Efficient de novo synthesis of resveratrol by metabolically engineered *Escherichia coli*. J Ind Microbiol Biotechnol.

[38] Chong Y, Yan A, Yang X, Cai Y, Chen J (2012). An optimum fermentation model established by genetic algorithm for biotransformation from crude polydatin to resveratrol. Appl Biochem Biotechnol.

[39] Park SR, Yoon JA, Paik JH, Park JW, Jung WS, Ban Y-H, Kim EJ, Yoo YJ, Han AR, Yoon YJ (2009). Engineering of plant-specific phenylpropanoids biosynthesis in *Streptomyces venezuelae*. J Biotechnol.

[40] Ni J, Tao F, Wang Y, Yao F, Xu P (2016). A photoautotrophic platform for the sustainable production of valuable plant natural products from CO_2_. Green Chem.

[41] Gaspar P, Dudnik A, Neves AR, Forster J. Engineering *Lactococcus lactis* for stilbene production. In: Presented at the 28th International Conference on Polyphenols. 2016.

[42] Shin SY, Jung SM, Kim MD, Han NS, Seo JH (2012). Production of resveratrol from tyrosine in metabolically engineered *Saccharomyces cerevisiae*. Enzyme Microb Technol.

[43] Palmer CM, Miller KK, Nguyen A, Alper HS (2020). Engineering 4-coumaroyl-CoA derived polyketide production in *Yarrowia Lipolytica* through a β-oxidation mediated strategy. Metab Eng.

[44] Katsuyama Y, Funa N, Horinouchi S (2007). Precursor-directed biosynthesis of stilbene methyl ethers in *Escherichia coli*. Biotechnol J.

[45] Westman JO, Franzén CJ (2015). Current progress in high cell density yeast bioprocesses for bioethanol production. Biotechnol J.

[46] Tang R, Weng C, Peng X, Han Y (2020). Metabolic engineering of *Cupriavidus necator* H16 for improved chemoautotrophic growth and PHB production under oxygen-limiting conditions. Metab Eng.

[47] Wang X, Luo H, Wang Y, Wang Y, Tu T, Qin X, Su X, Huang H, Bai Y, Yao B, Zhang J (2022). Direct conversion of carbon dioxide to glucose using metabolically engineered *Cupriavidus necator*. Bioresour Technol.

