# Supplementary material for: Carbon dioxide valorization into resveratrol via lithoautotrophic fermentation using engineered Cupriavidus necator H16
Source: Microb Cell Fact. 2024 Apr 27;23:122. doi: 10.1186/s12934-024-02398-x (PMC11055273; doi:10.1186/s12934-024-02398-x)
Supplement: Supplementary file 1 — Supplementary Material 1 [file 12934_2024_2398_MOESM1_ESM.docx]

**Additional file 1**

**Carbon dioxide valorization into resveratrol via lithoautotrophic fermentation using engineered *Cupriavidus necator* H16**

Yongjae Jang^1^, Yeon Ji Lee^1^, Gyeongtaek Gong^1,2^, Sun-Mi Lee^1,2^, Youngsoon Um^1,2^, Kyoung Heon Kim^3^, Ja Kyong Ko^1,2,^*

^1^Clean Energy Research Center, Korea Institute of Science and Technology (KIST), Seoul, 02792, Republic of Korea

^2^Division of Energy and Environment Technology, KIST School, University of Science and Technology, Seoul 02792, Republic of Korea

^3^Department of Biotechnology, Korea University, Seoul 02841, Republic of Korea

* Correspondence to J.K. Ko: [jkko@kist.re.kr](mailto:jkko@kist.re.kr)

**Table S1.** List of primers used in this study.

| **Name** | **Sequence^a^** |
| --- | --- |
| △phaCAB-F | GTGATCGCCATCATCAGCGCC |
| △phaCAB-R | CGTAGAGCCTACGGTTCGGATAC |
| phaC1-upstream-F | CGAGCTCGTGATCGCCATCATCAGCGCC |
| phaC1-upstream-R | CAGGCCGGCAGGGATTTGATTGTCTCTCTGCCGTCACTATT |
| phaB1-downstream-F | GACAATCAAATCCCTGCCGGCCTGGTTCAAC |
| phaB1-downstream-R | TGCTCTAGACGTAGAGCCTACGGTTCGGATAC |
| pBAD-insert-F | AAATGTACATTATGACAACTTGACGGCTACATCAT |
| pBAD-insert-R | AAAGAGCTCTCCTTCTTAAAAGATCTTTTGAATTCCCAA |
| FjTAL-F | AAAGATCTTTTAAGAAGGAGAGCTCATGAATACCATTAATGAGTACCTGAGC |
| FjTAL-R | CGAGGTCGACGGTATCGATAAGCTTTCAGTTATTGATCAGGTGGTCCTTC |
| VvSTS-F | TTCAAAAGATCTTTTAAGAAGGAGAGCTCATGGCCTCGGTGGAAGAAATC |
| VvSTS-R | CCCCTCGAGGTCGACGGTATCGATAAGCTTTCAGTTGGTCACCATCGGGAT |
| STS-TAL-F | AGGAGAGCTCCACCGCGGTGGCGGCCGCTCTAGAACTAGTATGGCCTCG  GTGGAAGAAATCCG |
| STS-TAL-R | CGGGCCCCCCCTCGAGGTCGACGGTATCGATAAGCTTTCAGTTATTGAT  CAGGTGGTCCTTC |
| STS-TAL-4CL-F | CTAGACTAGTATGGCCTCGGTGGAAGAAATCCGC |
| STS-TAL-4CL-R | CCCAAGCTTTCACAGGCCGTTCGCCAGCTTC |
| STS-TAL-4CL-ACC1-F | CGGCCTGTGAACAACCAAAGGAGGACAACCGTGTCAGTCGAGACTAGGAAGATCACCAAG |
| STS-TAL-4CL-ACC1-R | CCCAAGCTTTTACTTGATCTCGAGGAGAACAACGCCC |
| STS-TAL-4CL-ACC2-F | AAGGGCGTTGTTCTCCTCGAGATCAAGTAAACAACCAAAGGAGGACAACCATGACCATTTCCTCACCTTTGATTGACGT |
| STS-TAL-4CL-ACC2-R | AAGGGCGTTGTTCTCCTCGAGATCAAGTAAACAACCAAAGGAGGACAACCATGACCATTTCCTCACCTTTGATTGACGT |
| STS-TAL-4CL-ACC2-STS-F | CGACTGAGCCTTTCGTTTTATTTGGGTACCTTACAGTGGCATGTTGCCGTGCTTG |
| STS-TAL-4CL-ACC2-STS-F | GCTCGCAAGCACGGCAACATGCCACTGTAAACAACCAAAGGAGGACAACCATGGCCTCGGTGGAAGAAATC |
| STS-TAL-4CL-ACC2-STS-R | CCCAGTCTTTCGACTGAGCCTTTCGTTTTATTTGGGGTACCTCAGTTGGTCACCATCGGGAT |

^a^Restriction sites are underlined

**Table S2.** List of the gene sequences of *_Vv_*STS*,* *_Fj_*TAL, *_At_*4CL and *_Cg_*ACC used for the construction of recombinant plasmids.

| **Name** | **Sequence^a^** |
| --- | --- |
| *_Vv_STS* | ATGGCCTCGGTGGAAGAAATCCGCAACGCCCAACGCGCGAAAGGCCCGGCGACGATTCTGGCCATTGGCACCGCCACCCCGGACCACTGCGTGTATCAATCGGACTATGCCGACTATTATTTCCGCGTGACCAAATCGGAGCACATGAGCGAACTGAAGAAGAAGTTCAACCGCATCTGCGACAAGTCGATGATTAAGAAGCGCTATATCCATCTGACCGAAGAAATGCTGGAAGAACACCCGAACATCGGCGCGTATATGGCGCCGTCGCTGAACATCCGCCAGGAAATCATCACCGCCGAAGTGCCGAAGCTGGGCAAGGAAGCCGCCCTGAAGGCCCTGAAGGAATGGGGCCAGCCGAAGTCGAAGATCACCCATCTGGTGTTCTGCACCGCCAGCGGCGTGGAAATGCCGGGCGCGGACTATAAACTGGCCAACCTGCTGGGCCTGGAAACCTCGGTGCGCCGCGTGATGCTGTATCATCAAGGCTGCTATGCCGGCGGCACCGTGCTGCGTACCGCGAAGGACCTGGCCGAAAACAACGCCGGCGCGCGCGTGCTGGTGGTGTGCTCGGAAATCACCGTGGTGACCTTCCGTGGTCCGTCGGAAGACGCCCTGGACTCGCTGGTGGGTCAAGCCCTGTTCGGCGACGGCTCGGCGGCCGTGATTGTGGGCTCGGACCCGGACGTGTCGATTGAACGTCCGCTGTTCCAACTGGTGTCGGCGGCCCAAACCTTCATCCCGAACTCGGCGGGCGCGATTGCCGGTAACCTGCGTGAAGTGGGCCTGACCTTCCATCTGTGGCCGAACGTGCCGACCCTGATTTCGGAAAACGTGGAAAAGTGCCTGACGCAGGCGTTCGACCCGCTGGGCATTTCGGACTGGAACTCGCTGTTCTGGATTGCCCATCCGGGTGGCCCGGCCATTCTGGACGCCGTGGAAGCCAAACTGAACCTGGACAAAAAGAAACTGGAAGCCACCCGCCATGTGCTGTCGGAATATGGCAATATGTCGTCGGCCTGCGTGCTGTTCATCCTGGACGAAATGCGCAAAAAGTCGCATAAGGGCGAAAAGGCCACCACCGGGGAAGGTCTGGATTGGGGCGTGCTGTTCGGCTTCGGCCCCGGCCTGACCATCGAAACCGTGGTGCTGCATTCGATCCCGATGGTGACCAACTGA |
| *_Fj_TAL* | ATGAATACCATTAATGAGTACCTGAGCCTGGAGGAGTTCGAGGCGATCATCTTCGGCAACCAGAAGGTGACGATCAGCGACGTGGTGGTGAACCGCGTGAATGAGTCGTTCAATTTCCTGAAGGAGTTCAGCGGCAACAAGGTGATTTACGGCGTGAATACCGGCTTCGGCCCGATGGCCCAGTACCGCATTAAAGAGTCGGACCAGATTCAGCTCCAGTACAATCTGATTCGCTCGCATTCGAGCGGCACCGGCAAACCGCTGAGCCCGGTGTGCGCCAAAGCCGCGATCCTGGCCCGCCTGAATACCCTGAGCCTGGGTAATTCGGGCGTGCATCCGTCGGTCATCAACCTGATGTCGGAGCTGATCAACAAGGATATCACGCCGCTGATTTTCGAGCATGGCGGTGTGGGCGCGTCGGGCGATCTGGTGCAGCTGTCGCATCTGGCCCTGGTGCTGATTGGCGAGGGTGAGGTGTTTTACAAGGGCGAGCGCCGTCCGACCCCGGAGGTGTTCGAGATCGAGGGTCTGAAGCCGATTCAGGTGGAGATCCGCGAGGGCCTGGCGCTGATCAATGGCACCAGCGTCATGACCGGCATTGGCGTGGTGAATGTGTACCACGCGAAGAAGCTGCTGGATTGGAGCCTGAAGTCGAGCTGCGCGATCAACGAGCTGGTGCAGGCGTACGATGACCATTTTAGCGCCGAGCTGAACCAGACCAAGCGCCATAAGGGCCAGCAGGAGATCGCCCTGAAGATGCGCCAGAACCTGTCGGATTCGACCCTGATCCGCAAGCGCGAGGATCATCTGTACAGCGGCGAGAATACCGAGGAAATCTTCAAGGAGAAGGTGCAGGAGTACTACTCGCTGCGCTGCGTGCCGCAGATCCTCGGCCCGGTGCTGGAGACCATCAATAACGTGGCCTCGATCCTGGAGGACGAGTTCAATTCGGCCAATGACAACCCGATCATCGACGTGAAGAATCAGCATGTGTATCACGGCGGCAACTTCCACGGCGATTACATCTCGCTGGAGATGGACAAGCTGAAGATCGTGATCACCAAGCTGACCATGCTGGCCGAGCGCCAGCTGAATTACCTGCTGAATAGCAAGATCAATGAGCTGCTGCCGCCGTTCGTGAATCTGGGCACCCTGGGCTTCAACTTCGGCATGCAGGGCGTGCAGTTCACCGCCACCTCGACCACCGCCGAGTCGCAGATGCTGTCGAACCCGATGTACGTGCACTCGATCCCGAACAATAACGACAATCAGGACATCGTGTCGATGGGCACCAACAGCGCCGTGATCACCAGCAAGGTCATCGAGAACGCCTTCGAAGTGCTGGCCATCGAGATGATCACCATCGTGCAGGCCATCGACTACCTGGGCCAGAAGGATAAGATCAGCAGCGTGTCGAAGAAGTGGTACGACGAGATCCGCAATATCATCCCGACCTTTAAGGAGGATCAGGTGATGTACCCGTTCGTGCAGAAGGTGAAGGACCACCTGATCAATAACTGA |
| *_At_4CL* | ATGGCCCCGCAGGAACAGGCGGTGAGCCAGGTCATGGAAAAGCAGTCGAACAATAACAATAGCGACGTCATCTTCCGCAGCAAGCTGCCGGACATCTATATCCCGAACCACCTGAGCCTGCACGACTACATCTTCCAGAACATCAGCGAGTTCGCGACGAAGCCGTGCCTGATCAACGGCCCGACGGGGCATGTGTACACGTACAGCGACGTCCATGTGATCAGCCGCCAGATCGCGGCGAACTTCCATAAGCTGGGCGTCAACCAGAACGACGTGGTCATGCTGCTGCTGCCGAATTGCCCGGAATTTGTGCTGAGCTTCCTGGCGGCCAGCTTCCGCGGGGCGACGGCGACCGCGGCGAACCCGTTCTTCACGCCGGCGGAAATCGCGAAGCAGGCGAAGGCGAGCAACACGAAGCTGATCATCACCGAAGCCCGCTACGTGGACAAGATCAAGCCGCTCCAGAACGACGACGGCGTCGTGATCGTCTGCATCGATGACAACGAAAGCGTGCCGATCCCGGAAGGCTGCCTGCGGTTCACGGAACTGACGCAGAGCACCACGGAAGCGAGCGAAGTCATCGATAGCGTGGAAATCAGCCCGGATGACGTCGTGGCGCTGCCGTACAGCAGCGGCACGACGGGCCTGCCGAAAGGCGTCATGCTGACGCACAAGGGCCTGGTCACGAGCGTCGCGCAGCAGGTCGATGGCGAGAACCCGAACCTGTACTTCCACAGCGACGATGTGATCCTGTGCGTGCTGCCGATGTTCCACATCTACGCGCTGAACTCGATCATGCTGTGCGGCCTGCGTGTCGGCGCGGCCATCCTGATCATGCCGAAATTCGAGATCAACCTGCTGCTGGAACTGATCCAACGGTGCAAGGTGACGGTGGCCCCGATGGTCCCACCAATTGTCTTGGCGATTGCGAAGAGTAGCGAGACAGAAAAGTATGATTTGAGCAGCATTCGTGTCGTGAAAAGCGGCGCGGCACCACTGGGTAAAGAACTGGAAGATGCGGTGAACGCGAAGTTCCCGAACGCGAAGCTGGGCCAAGGCTATGGCATGACCGAGGCCGGCCCGGTCCTGGCGATGAGCCTGGGCTTCGCGAAAGAACCGTTCCCGGTCAAGAGCGGCGCGTGCGGCACGGTCGTCCGCAACGCGGAGATGAAAATCGTCGATCCGGACACGGGCGACAGCCTGAGCCGCAACCAACCGGGCGAAATCTGCATCCGCGGCCACCAAATCATGAAGGGCTATCTGAACAACCCGGCGGCCACCGCCGAGACCATCGACAAAGACGGCTGGCTGCACACGGGCGACATCGGCCTGATCGACGACGACGACGAACTGTTCATCGTGGACCGCCTGAAGGAACTGATCAAGTACAAGGGCTTCCAAGTGGCCCCAGCCGAGCTGGAGGCGCTGCTGATCGGTCACCCGGACATCACCGACGTGGCGGTGGTCGCCATGAAAGAGGAGGCCGCGGGCGAGGTCCCGGTGGCGTTCGTGGTGAAATCGAAAGACAGCGAGCTGTCGGAAGACGACGTGAAGCAATTCGTGAGCAAGCAAGTGGTGTTCTACAAACGTATCAACAAAGTGTTCTTCACCGAGAGCATCCCAAAAGCGCCGAGCGGCAAAATCCTGCGTAAAGACCTGCGTGCGAAGCTGGCGAACGGCCTGTGA |
| *_Cg_ACC* | GTGTCAGTCGAGACTAGGAAGATCACCAAGGTTCTTGTCGCTAACCGTGGTGAGATTGCAATCCGCGTGTTCCGTGCAGCTCGAGATGAAGGCATCGGATCTGTCGCCGTCTACGCAGAGCCAGATGCAGATGCACCATTCGTGTCATATGCAGACGAGGCTTTTGCCCTCGGTGGCCAAACATCCGCTGAGTCCTACCTTGTCATTGACAAGATCATCGATGCGGCCCGCAAGTCCGGCGCCGACGCCATCCACCCCGGCTACGGCTTCCTCGCAGAAAACGCTGACTTCGCAGAAGCAGTCATCAACGAAGGCCTGATCTGGATTGGACCTTCACCTGAGTCCATCCGCTCCCTCGGCGACAAGGTCACCGCTCGCCACATCGCAGATACCGCCAAGGCTCCAATGGCTCCTGGCACCAAGGAACCAGTAAAAGACGCAGCAGAAGTTGTGGCTTTCGCTGAAGAATTCGGTCTCCCAATCGCCATCAAGGCAGCTTTCGGTGGCGGCGGACGTGGCATGAAGGTTGCCTACAAGATGGAAGAAGTCGCTGACCTCTTCGAGTCCGCAACCCGTGAAGCAACCGCAGCGTTCGGCCGCGGCGAGTGCTTCGTGGAGCGCTACCTGGACAAGGCACGCCACGTTGAGGCTCAGGTCATCGCCGATAAGCACGGCAACGTTGTTGTCGCCGGAACCCGTGACTGCTCCCTGCAGCGCCGTTTCCAGAAGCTCGTCGAAGAAGCACCAGCACCATTCCTCACCGATGACCAGCGCGAGCGTCTCCACTCCTCCGCGAAGGCTATCTGTAAGGAAGCTGGCTACTACGGTGCAGGCACCGTTGAGTACCTCGTTGGCTCCGACGGCCTGATCTCCTTCCTCGAGGTCAACACCCGCCTCCAGGTGGAACACCCAGTCACCGAAGAGACCACCGGCATCGACCTGGTCCGCGAAATGTTCCGCATCGCAGAAGGCCACGAGCTCTCCATCAAGGAAGATCCAGCTCCACGCGGCCACGCATTCGAGTTCCGCATCAACGGCGAAGACGCTGGCTCCAACTTCATGCCTGCACCAGGCAAGATCACCAGCTACCGCGAGCCACAGGGCCCAGGCGTCCGCATGGACTCCGGTGTCGTTGAAGGTTCCGAAATCTCCGGACAGTTCGACTCCATGCTGGCAAAGCTGATCGTTTGGGGCGACACCCGCGAGCAGGCTCTCCAGCGCTCCCGCCGTGCACTTGCAGAGTACGTTGTCGAGGGCATGCCAACCGTTATCCCATTCCACCAGCACATCGTGGAAAACCCAGCATTCGTGGGCAACGACGAAGGCTTCGAGATCTACACCAAGTGGATCGAAGAGGTTTGGGATAACCCAATCGCACCTTACGTTGACGCTTCCGAGCTCGACGAAGATGAGGACAAGACCCCAGCACAGAAGGTTGTTGTGGAGATCAACGGCCGTCGCGTTGAGGTTGCACTCCCAGGCGATCTGGCACTCGGTGGCACCGCTGGTCCTAAGAAGAAGGCCAAGAAGCGTCGCGCAGGTGGTGCAAAGGCTGGCGTATCCGGCGATGCAGTGGCAGCTCCAATGCAGGGCACTGTCATCAAGGTCAACGTCGAAGAAGGCGCTGAAGTCAACGAAGGCGACACCGTTGTTGTCCTCGAGGCTATGAAGATGGAAAACCCTGTGAAGGCTCATAAGTCCGGAACCGTAACCGGCCTTACTGTCGCTGCAGGCGAGGGTGTCAACAAGGGCGTTGTTCTCCTCGAGATCAAGTAA**(*ACC1*)**ACAACCAAAGGAGGACAACC**(RBS)**ATGACCATTTCCTCACCTTTGATTGACGTCGCCAACCTTCCAGACATCAACACCACTGCCGGCAAGATCGCCGACCTTAAGGCTCGCCGCGCGGAAGCCCATTTCCCCATGGGTGAAAAGGCAGTAGAGAAGGTCCACGCTGCTGGACGCCTCACTGCCCGTGAGCGCTTGGATTACTTACTCGATGAGGGCTCCTTCATCGAGACCGATCAGCTGGCTCGCCACCGCACCACCGCTTTCGGCCTGGGCGCTAAGCGTCCTGCAACCGACGGCATCGTGACCGGCTGGGGCACCATTGATGGACGCGAAGTCTGCATCTTCTCGCAGGACGGCACCGTATTCGGTGGCGCGCTTGGTGAGGTGTACGGCGAAAAGATGATCAAGATCATGGAGCTGGCAATCGACACCGGCCGCCCATTGATCGGTCTTTACGAAGGCGCTGGCGCTCGTATTCAGGACGGCGCTGTCTCCCTGGACTTCATTTCCCAGACCTTCTACCAAAACATTCAGGCTTCTGGCGTTATCCCACAGATCTCCGTCATCATGGGCGCATGTGCAGGTGGCAACGCTTACGGCCCAGCTCTGACCGACTTCGTGGTCATGGTGGACAAGACCTCCAAGATGTTCGTTACCGGCCCAGACGTGATCAAGACCGTCACCGGCGAGGAAATCACCCAGGAAGAGCTTGGCGGAGCAACCACCCACATGGTGACCGCTGGTAACTCCCACTACACCGCTGCGACCGATGAGGAAGCACTGGATTGGGTACAGGACCTGGTGTCCTTCCTCCCATCCAACAATCGCTCCTACGCACCGATGGAAGACTTCGACGAGGAAGAAGGCGGCGTTGAAGAAAACATCACCGCTGACGATCTGAAGCTCGACGAGATCATCCCAGATTCCGCGACCGTTCCTTACGACGTCCGCGATGTCATCGAATGCCTCACCGACGATGGCGAATACCTGGAAATCCAGGCAGACCGCGCAGAAAACGTTGTTATTGCATTCGGCCGCATCGAAGGCCAGTCCGTTGGCTTTGTTGCCAACCAGCCAACCCAGTTCGCTGGCTGCCTGGACATCGACTCCTCTGAGAAGGCAGCTCGCTTCGTCCGCACCTGCGACGCGTTCAACATCCCAATCGTCATGCTTGTCGACGTCCCCGGCTTCCTCCCAGGCGCAGGCCAGGAGTACGGTGGCATTCTGCGTCGTGGCGCAAAGCTGCTCTACGCATACGGCGAAGCAACCGTTCCAAAGATCACCGTCACCATGCGTAAGGCTTACGGCGGAGCGTACTGCGTGATGGGTTCCAAGGGCTTGGGCTCTGACATCAACCTTGCATGGCCAACCGCACAGATCGCCGTCATGGGCGCTGCTGGCGCAGTTGGATTCATCTACCGCAAGGAGCTCATGGCAGCTGATGCCAAGGGCCTCGATACCGTAGCTCTGGCTAAGTCCTTCGAGCGCGAGTATGAAGACCACATGCTCAACCCGTACCACGCTGCAGAACGTGGCCTGATCGACGCCGTGATCCTGCCAAGCGAAACCCGCGGACAGATTTCCCGCAACCTTCGCCTGCTCAAGCACAAGAACGTCACTCGCCCTGCTCGCAAGCACGGCAACATGCCACTGTAA **(*ACC2*)** |


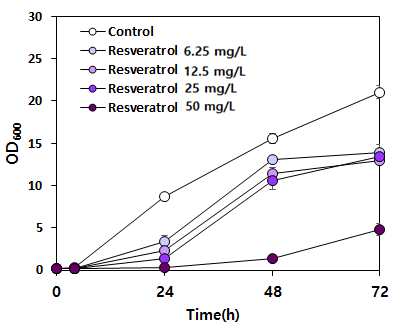


**Fig. S1.** Inhibitory effect of resveratrol on cell growth of *C. necator* H16 at various concentrations ranging from 0 to 50 mg/L. Cells were grown in MM medium containing 10 g/L fructose and 1 g/L (NH_4_)_2_SO_4_.


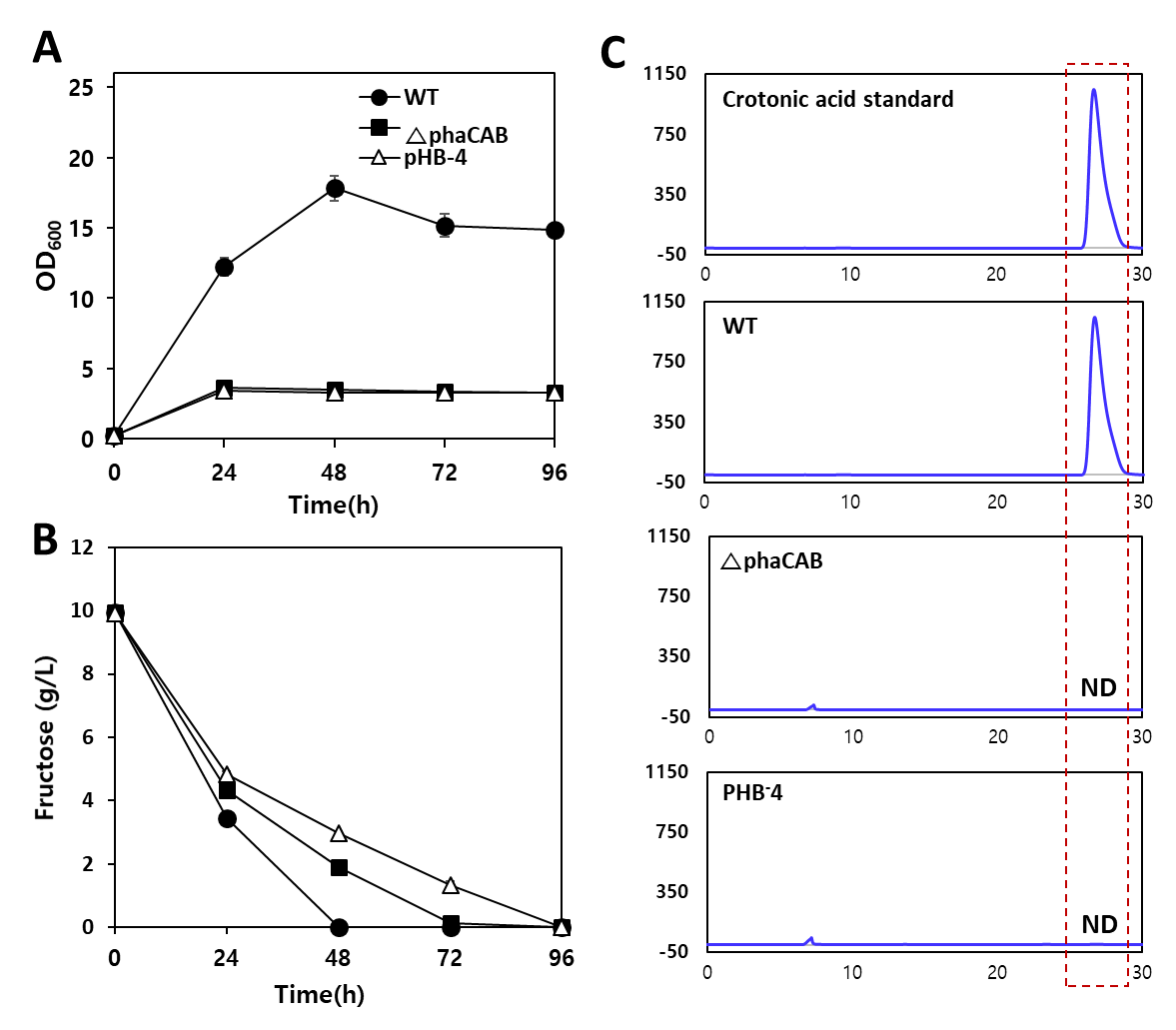


**Fig. S2**. The cell growth and PHB accumulation in *C. necator* H16 wild type (WT), PHB-negative △CAB, and PHB^-^4 strains during heterotrophic fermentation (10 g/L fructose). (A) Cell growth, (B) fructose consumption, and (C) crotonic acid peak in HPLC chromatograms to confirm the PHB occurrence.


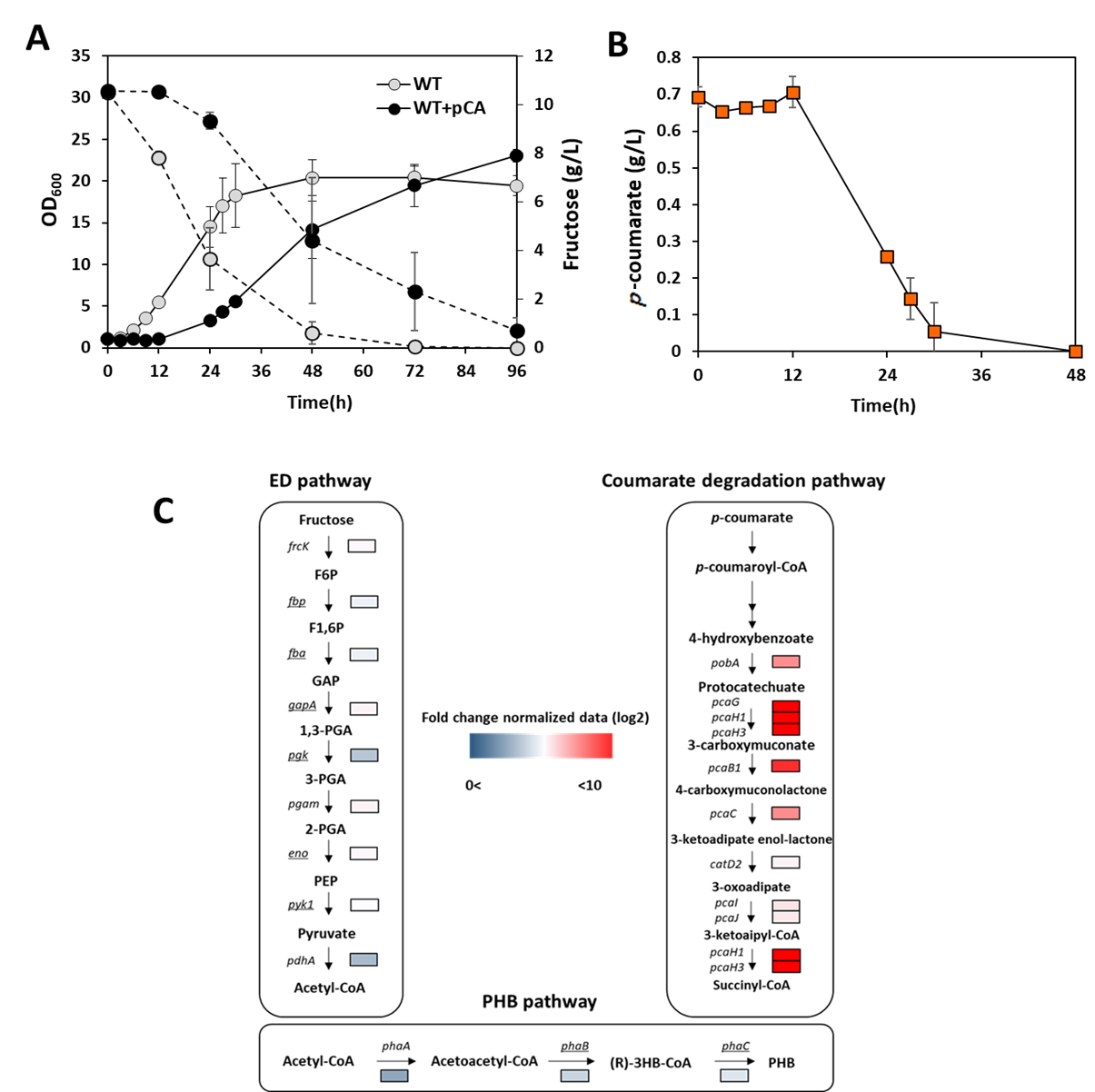


**Fig. S3.** Catabolism of *p*-coumaric acid in *C. necator* H16. RNA-seq samples were prepared after 20 h of aerobic fermentation in a minimal medium containing 10 g/L fructose with or without 1 g/L *p*-coumaric acid. (A) Profiles of the cell growth and fructose consumption, (B) *p*-coumarate utilization by *C. necator* H16. (C) Transcriptomic changes of genes involved in the putative *p*-coumarate degrading metabolism in *C. necator* H16. In transcriptomic comparative analysis, the cells grown in the presence of *p*-coumaric acid (WT+pCA; black circle) was the experimental group while the strain grown in only fructose (WT; grey circle) was the control group (Fold changes > 1.5, p value < 0.05).
